# Intraoperative Hypotension and Postoperative Newly Developed Cerebral Infarction in Patients With Aneurysmal Subarachnoid Hemorrhage: A Retrospective Cohort Study

**DOI:** 10.1111/cns.70156

**Published:** 2024-12-09

**Authors:** Min Zeng, Xueke Yin, Maoyao Zheng, Yue Ren, Shu Li, Xiaolin Chen, Yuming Peng

**Affiliations:** ^1^ Department of Anesthesiology Beijing Tiantan Hospital, Capital Medical University Beijing PR China; ^2^ Department of Neurosurgery Beijing Tiantan Hospital, Capital Medical University Beijing PR China; ^3^ Outcome Research Consortium Houston Texas USA

**Keywords:** aneurysmal subarachnoid hemorrhage, hypotension, newly developed cerebral infarction

## Abstract

**Aims:**

To investigate the association between intraoperative hypotension and newly developed cerebral infarction in patients with aneurysmal subarachnoid hemorrhage (aSAH) undergoing aneurysm clipping or coiling.

**Methods:**

The patients who had emergent clipping/coiling procedures for aSAH under general anesthesia were included. The major exposure was mean arterial pressure (MAP) below different absolute or relative thresholds characterized by area under curve (AUC), duration, and time‐weighted average (TWA) value. The outcome was newly developed cerebral infarction. The associations between MAP and newly developed cerebral infarction were adjusted by other risk factors. Odds ratio and 95% confidence interval were used to present the statistical difference.

**Results:**

A total of 1205 patients were included in the analysis. Of these, 260 patients (21.6%) developed new cerebral infarctions assessed by computed tomography. Patients with newly developed cerebral infarction had higher incidence of modified Fisher Scale (mFS) score 3 to 4 (80.0 vs. 69.1%, *p* < 0.01) and longer duration of anesthesia (4.3 vs. 3.9 h, *p* < 0.01). In the multivariate model, the AUC‐MAP (adjusted odds ratio: 1.00, 95% CI: 1.000 to 1.000, *p* = 0.02) and the TWA‐MAP (adjusted odds ratio: 1.01, 95% CI: 1.001 to 1.024, *p* = 0.04) of 20% decrease from baseline were closely associated with the newly developed cerebral infarction.

**Conclusions:**

Mean arterial pressure decreased 20% from baseline value were independently associated with postoperative newly developed cerebral infarction in patients with aSAH.

## Graphical abstract

After aneurysmal subarachnoid hemorrhage, the cerebral autoregulation is destroyed, and the cerebral blood flow is dependent on blood pressure. Reliable recommendations on periprocedural blood pressure target are still lacking and remains inconclusive. This study aimed to investigate the association between intraoperative hypotension and newly developed cerebral infarction.

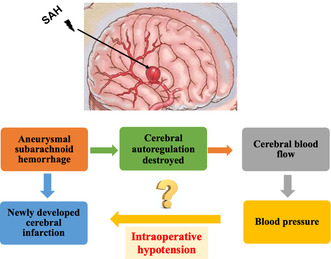

## 1 Introduction

Aneurysmal subarachnoid hemorrhage (aSAH) is a potentially fatal neurological disease. Despite considerable treatment improvements over the past decades, mortality and disability rates for aSAH patients remain high [1, 2]. The reported mortality rate is 40%–45% at 30 days after bleeding and a third of the survivors have extracranial sequelae [3, 4, 5]. Besides, posttreatment cerebral infarction was strongly associated with poor outcomes. Newly developed cerebral infarction was the common complications associated with bad outcomes [6].

It has been suggested that the risk factors for postoperative cerebral infarction after aSAH included poor grade, intracranial hypertension, global impairment of cerebral perfusion, symptomatic vasospasm, larger aneurysm domes, and posterior circulation ruptured aneurysms [7, 8]. After aSAH, the cerebral autoregulation is destroyed, and the cerebral blood flow is dependent on blood pressure. It is critical to balance the risk of hypertension‐related rebleeding and hypotension‐related cerebral ischemia for aSAH with patients' general anesthesia [9, 10]. Previously, only a few case series or small‐sample clinical trials attempted to assess the association between intraoperative blood pressure and new onset cerebral infarctions [11, 12, 13], and there is still lacking reliable recommendations on target for intraoperative blood pressure [1].

Thus, we conducted a retrospective cohort study and aimed to investigate the association between intraoperative hypotension and newly developed cerebral infarction in aSAH patients undergoing aneurysm clipping or coiling.

## 2 Methods

### 2.1 Participants

The retrospective study adhered to Strengthening the Reporting of Cohort Studies in Surgery (STROCSS) criteria [14]. The Ethics Committee approved this study and waived the requirement for informed consent.

We consecutively included patients who had emergent clipping/coiling procedures for aSAH under general anesthesia between November 1, 2015 and December 31, 2021 in Beijing Tiantan Hospital, Capital Medical University. The exclusion criteria were as follows: hospital admission later than 48 h after ictus; no aneurysm surgical treatment; no data on intraoperative blood pressure or data missed for > 10 consecutive minutes; incomplete computed tomography (CT) scan (missing preoperative or/and postoperative CT scan).

### 2.2 Data Collection

From the electronic medical records, we obtained the following variables: demographic, clinical (American Society of Anesthesiologists physical status, coexisting medical condition, preoperative medication), laboratory, radiographic, and treatment‐related information (type of surgery and anesthesia, use of vasopressors, the total volume of fluid input and output, and duration of surgery and anesthesia, postoperative delayed tracheal extubation, length of hospital stay, medical cost, and clinical outcome at discharge).

The severity of subarachnoid hemorrhage is commonly assessed using the Hunt‐Hess and World Federation of Neurosurgery Scales (WFNS). The Hunt‐Hess scale has five grades, ranging from minimally symptomatic to coma, while the WFNS scale has five grades, ranging from normal consciousness to major impairments and coma. The modified Fisher Scale (mFS) is a method of grading severity of subarachnoid hemorrhage from the rupture of an intracranial aneurysm, ranging from mild (Grade 1) to severe (Grade 4). Patients classified as WFNS Grade 4 or 5, Hunt‐Hess Grade 4 or 5, and mFS Grade 3 or 4 are in poor clinical status [15]. The modified Rankin Scale (mRS) is a clinician‐reported measure of stroke disability scored on a scale of 0 to 6, 0 indicating no symptoms of disability, 5 indicating severe disability, and 6 indicating death. Favorable outcomes were defined as the mRS score of 0 to 2, while poor outcomes were defined as of 3–6 [16]. Two investigators independently collected and extracted data. Differences were resolved by consensus among the team members or, when needed, by a senior researcher.

### 2.3 Newly Developed Cerebral Infarction

In this study, newly developed cerebral infarction was evaluated by reviewing the preoperative and postoperative CT scan, which was defined as a newly detected hypodensity on the CT scan during early postoperative period whereas invisible on admission and unrelated to the surgical approach. Hypodensity associated with surgical procedures was due to the injury of surgical approach, not perioperative hypotension. So, the hypodensity associated with surgical procedures was not regarded as newly developed cerebral infarction [6].

### 2.4 Blood Pressure Collection and Processing

Intraoperative pressures data were extracted from the Anesthesia Information Management System (AIMS, version 5.0, Wangfeng Mingyue Ltd., China). The measurement system included built‐in artifact filtering, which reduced the resonance effects of the tubing system. The invasive pressures were recorded at intervals of 10 s while noninvasive pressure with an oscillometric brachial cuff at intervals of 5 min.

Blood pressure data were excluded according to the criteria published in the previous study [17]. For each subject, mean arterial pressure (MAP) of 65, 70, 75 mmHg or 20%, 30%, 40% decrease from baseline value before anesthesia induction were defined as the levels of intraoperative hypotension. The area under curve (AUC) of each level of hypotension was defined as the cumulative sums of the area using the trapezoid rule and reported in unit of mmHg times minutes. Calculation of the area under with specific level started and ended when MAP was below and greater than this level. The duration of hypotension was calculated as the cumulative duration below each threshold in minutes. The time‐weighted average (TWA) of MAP was derived by dividing the area under the level by the anesthesia duration [18, 19].

### 2.5 Statistical Analysis

Parameters were compared between patients whether experienced postoperative newly developed cerebral infarction or not. Categorical variables are presented as counts (percentages) and analyzed by using either the *χ* ^2^ or Fisher's exact test.

Continuous variables including body mass index, duration of anesthesia, length of stay, and medical costs were tested for normality using a quantile–quantile plot of residuals and the Shapiro–Wilk test. Continuous variables are presented as the mean (standard deviation, SD) or the median (interquartile range, IQR). Normal distributed variables were compared by Student's *t*‐test and Mann–Whitney test was applied if not.

The preoperative and intraoperative variables were chosen as potential confounding factors for the association, based on the univariate analyses (*p* < 0.05). The diagnostics of multivariate regression model were tested by Hosmer–Lemeshow goodness of fit test. The interaction was detected using interaction terms in the regression model, and a variance inflation factor of < 5 was considered as an absence of multicollinearity. In addition, we performed a post hoc subgroup analysis to evaluate the association between hypotension and newly developed cerebral infarction according to age, ASA physical status classification, and anesthesia maintenance method.

The results are presented as odds ratios (OR) or adjusted odds ratios (aOR) with 95% confidence intervals (CIs). For all outcomes, a *p* < 0.05 was considered statistically significant. All statistical analyses were performed using the Stata/SE 16.0 (Stata Corp, TX, USA).

## 3 Results

**FIGURE 1:**
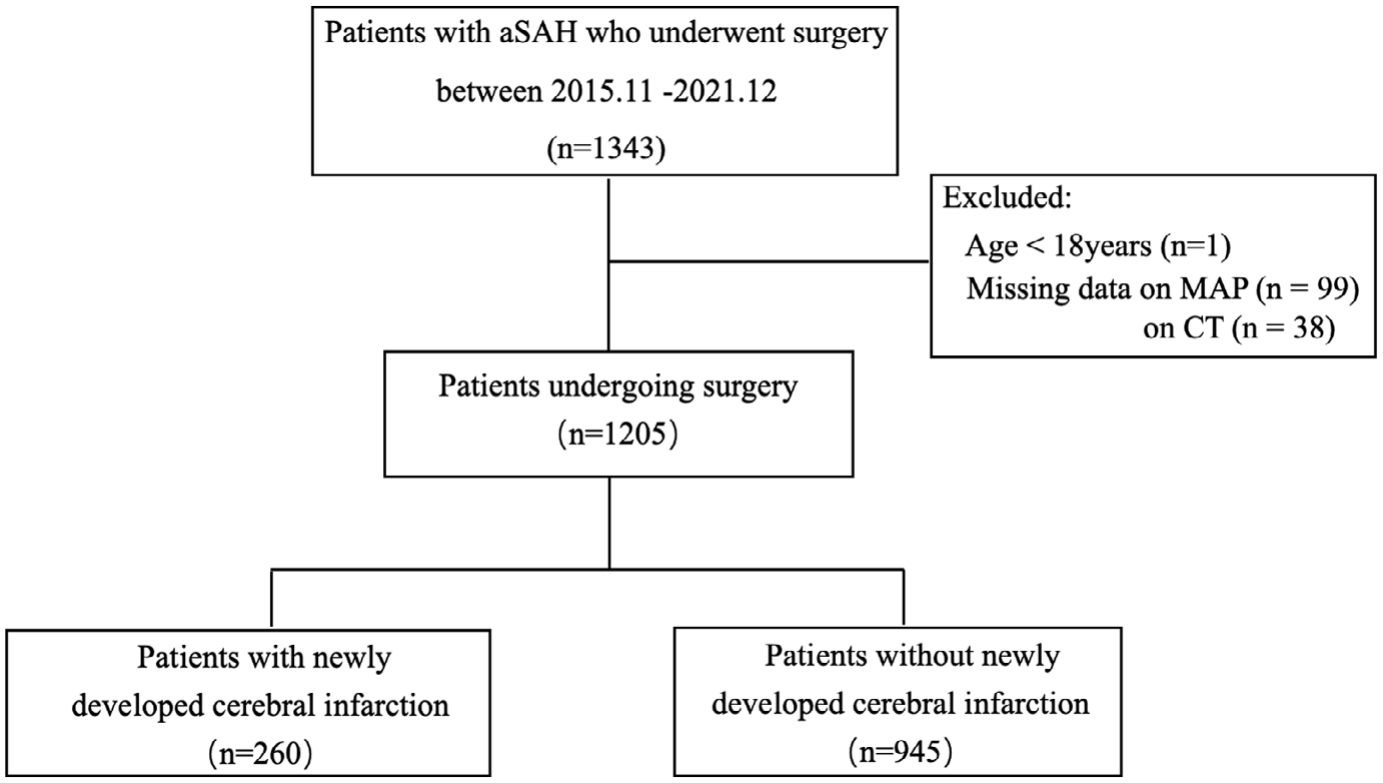
Study flowchart.

A flowchart of this study is shown in Figure 1. A total of 1343 aSAH patients who underwent surgery between November 1, 2015, and December 31, 2021. Finally, 1205 patients were included in the analysis. Ruptured aneurysms were clipped in 673 patients and coiled in 532 patients. Newly developed cerebral infarctions were found in 260 patients (21.6%) evaluated by brain CT scan at a median of 2 days. Frontal and temporal lobe, and basal ganglia area were frequently involved (see Table 1).

**TABLE 1:** Characteristics of postoperative cerebral infarction.

	Newly onset cerebral infarction
	*n* = 260
Median CT time—days	2 (1–5)
Location	
Frontal	131 (50.4)
Temporal	49 (18.8)
Occipital	21 (8.1)
Parietal	34 (13.1)
Cerebellum	27 (10.4)
Brainstem	11 (4.2)
Basal ganglia area	89 (34.2)
Thalamus	26 (10.0)
Corpus callosum	28 (10.8)
Others^a^	36 (13.8)

*Note:* Values are presented as median (interquartile range), or number (%).

Abbreviation: IQR, interquartile range.

^a^ Others indicate, lateral ventricle, centrum semiovale, capsula interna, corona radiata, insula, and brachium pontis.

Baseline characteristics and surgery‐related parameters are listed in Tables 2 and 3. Patients with newly developed cerebral infarction had higher incidence of mFS of 3 to 4 (80.0 vs. 69.1%, *p* < 0.01). The mean duration of anesthesia was significantly longer in patients who experienced newly developed cerebral infarction (4.3 vs. 3.9 h, *p* < 0.001). Patients with newly developed cerebral infarction had a significantly higher proportion of delayed tracheal intubation (25.2 vs. 14.2%, *p* < 0.001), a significantly longer hospital stay (15.0 vs. 12.0 days, *p* < 0.001), higher incidence of adverse cardiac events (21.2 vs. 12.7%, *p* < 0.001), pulmonary infection (41.9 vs. 24.4%, *p* < 0.001), venous thrombosis (36.2 vs. 22.6%, *p* < 0.001), and hypoproteinemia (30.8 vs. 21.3%, *p* = 0.001) than those patients who did not. In addition, the incidence of a favorable outcome at discharge (29.2 vs. 62.9%, *p* < 0.001) and at 3 months (56.3 vs. 87.8%, *p* < 0.001) were significantly lower in patients with newly developed cerebral infarction (Table 3). In the multivariate model for newly developed cerebral infarction, the mFS score of 3 to 4 (OR, 1.61; 95% CI: 1.14–2.28, *p* = 0.01) and the duration of anesthesia per hour (OR, 1.00; 95% CI: 1.00–1.00, *p* = 0.02) were indicated to be statistical significance without multicollinearity (Table 4).

**TABLE 2:** Characteristics of baseline parameters.

Variable	All patients	Newly developed cerebral infarction	Without newly developed cerebral infarction	*p*
	*N* = 1205	*n* = 260	*n* = 945	
Mean age, years	55.2 (10.9)	55.2 (11.3)	55.17 (10.9)	0.98
Age > 70	107 (8.9)	30 (11.5)	77 (8.1)	0.09
Female sex	737 (61.2)	157 (60.4)	580 (61.4)	0.77
BMI	24.5 (22.7–27.1)	25.1 (22.8–27.5)	24.3 (22.7–7.0)	0.65
Preoperative comorbid conditions				
Hypertension	756 (62.7)	168 (64.6)	588 (62.2)	0.48
Coronary heart disease	119 (9.9)	30 (11.5)	89 (9.4)	0.31
Arrythmia	70 (5.8)	17 (6.5)	53 (5.6)	0.57
Diabetes	90 (7.5)	26 (10.0)	64 (6.8)	0.08
Chronic pulmonary disease	16 (1.3)	3 (1.2)	13 (1.1)	0.78
Hemorrhagic stroke	17 (1.4)	2 (0.8)	15 (1.6)	0.32
Ischemic stroke	71 (5.9)	19 (7.3)	52 (5.5)	0.27
Smoking	331 (27.5)	80 (29.6)	251 (26.8)	0.37
Drinking	211 (17.5)	50 (18.5)	161 (17.2)	0.62
Medication use prior to admission				
Antihypertensive drug	213 (17.7)	47 (18.1)	166 (17.6)	0.85
Antiplatelet drugs	64 (5.3)	17 (6.5)	47 (5.0)	0.32
β‐blocking agents	16 (1.3)	2 (0.8)	14 (1.5)	0.37
Calcium antagonist	127 (10.5)	31 (11.9)	96 (10.2)	0.41
ACEI	63 (5.2)	14 (5.4)	49 (5.2)	0.90
Preoperative assessments				
ASA physical status				0.23
II–III	1102 (91.5)	233 (89.6)	869 (92)	
IV–V	103 (8.5)	27 (10.4)	76 (8.0)	
OAA/S scale				0.00
≤ 2	245 (20.3)	74 (28.5)	171 (18.1)	
> 2	960 (79.7)	186 (71.5)	774 (81.9)	
Modified Fisher Scale				0.00
Grade 0–2	344 (28.5)	52 (20.0)	292 (30.9)	
Grade 3–4	777 (71.4)	208 (80.0)	653 (69.1)	
WFNS scale				0.00
Grade 1–3	932 (77.3)	195 (71.4)	750 (80.5)	
Grade 4–5	273 (22.7)	78 (28.6)	182 (19.5)	
Locations of ruptured aneurysm				0.18
Anterior circulation ^a^	967 (80.2)	201 (77.3)	766 (81.1)	
Posterior circulation ^b^	238 (19.8)	59 (22.7)	179 (18.9)	
Time from symptom onset to surgery, days	2 (1–4)	2 (1–4)	2 (1–4)	0.12

*Note:* Values are presented as means (standard deviations), median (interquartile range), or number (%). ^a^: Anterior circulation: aneurysm at anterior cerebral artery, middle cerebral artery, anterior communication artery, anterior choroidal artery, internal carotid artery, ophthalmic artery, and posterior communication artery; ^b^: posterior circulation: aneurysm at posterior cerebral artery, superior cerebellar artery and posterior inferior cerebellar artery.

Abbreviations: ACEI, angiotensin converting enzyme inhibitors; ASA, American Society of Anesthesiologists; BMI, body mass index; OAA/S scale, observer assessment of alertness/sedation scale; WFNS, world federation of neurosurgery societies scale.

**TABLE 3:** Anesthesia‐ and surgery‐related characteristics.

Variable	All patients	Newly developed cerebral infarction	Without newly developed cerebral infarction	*p*
	*N* = 1205	*n* = 260	*n* = 945	
Methods of treatment				0.00
Neurosurgical Clipping	673 (55.9)	169 (65.0)	504 (53.3)	
Endovascular Coiling	532 (44.1)	91 (35.0)	441 (46.7)	
Type of anesthesia				0.00
TIVA	581 (48.2)	105 (40.4)	476 (50.4)	
CIVIA	624 (51.8)	155 (59.6)	469 (49.6)	
Mean duration of anesthesia, hours	4.0 (3.0–5.1)	4.3 (3.4–5.5)	3.9 (2.8–5.0)	0.00
Intraoperative medication				
Vasopressor	288 (23.9)	67 (25.8)	221 (23.4)	0.43
Antihypertensive medication	204 (16.9)	54 (20.8)	150 (15.9)	0.06
Retention of endotracheal catheter	201 (16.7)	68 (25.2)	133 (14.2)	0.00
Postoperative complications				
Adverse cardiac events	175 (14.5)	55 (21.2)	120 (12.7)	0.00
Pulmonary infection	340 (28.2)	109 (41.9)	231 (24.4)	0.00
Pulmonary edema	19 (1.6)	6 (2.3)	13 (1.4)	0.29
Venous thrombosis	308 (25.6)	94 (36.2)	214 (22.6)	0.00
Hypoproteinemia	281 (23.3)	80 (30.8)	201 (21.3)	0.00
Anemia	288 (23.9)	78 (30.0)	210 (22.2)	0.01
Stress ulcer	195 (16.2)	51 (19.6)	144 (15.2)	0.20
Liver injury	293 (24.3)	88 (33.8)	205 (21.7)	0.00
Kidney injury	56 (4.6)	21 (8.1)	35 (3.7)	0.00
Discharge mRS				0.00
≤ 2	670 (55.6)	76 (29.2)	594 (62.9)	
> 2	535 (44.4)	184 (70.8)	351 (37.1)	
In‐hospital mortality	17 (1.4)	5 (1.9)	12 (1.3)	0.43
Mean LOS, days	13.0 (9.0–17.0)	15.0 (11.0–20.0)	12.0 (8.0–16.0)	0.00
Mean medical costs, ×10^3^	11.4 (7.7–17.0)	11.7 (7.9–17.5)	11.3 (7.6–16.7)	0.28
3 months mRS				0.00
≤ 2	723 (81.5)	99 (56.3)	624 (87.8)	
> 2	164 (18.5)	77 (43.8)	87 (12.2)	
3 months mortality	22 (2.5)	8 (4.5)	14 (2.0)	0.05

*Note:* Values are presented as median (interquartile range) or number (%).

Abbreviations: CIVIA, combined intravenous–inhalation anesthesia; LOS, length of hospital stay; mRS, modified ranking scale; TIVA, total intravenous anesthesia.

**TABLE 4:** Risk factors for newly developed cerebral infarction in univariate and multivariate analysis.

Predictor variables	Univariate analysis			Multivariate analysis		
	OR	95% CI	*p*	aOR	95% CI	*p*
OAA/S scale > 2	1.80	1.31–2.47	0.00	1.54	0.86–2.76	0.15
Modified Fisher Scale, Grade 3–4	1.79	1.28–2.50	0.00	1.61	1.14–2.28	0.01
WFNS scale, Grade 1–3	1.65	1.21–2.24	0.00	1.03	0.58–1.82	0.92
Neurosurgical clipping	1.63	1.22–2.16	0.00	1.34	0.82–2.20	0.25
TIVA	1.50	1.13–1.98	0.00	0.96	0.60–1.52	0.85
Mean duration of anesthesia (h)	1.20	1.10–1.31	0.00	1.00	1.00–1.00	0.02

*Note:* The variance inflation factor (VIF) method was used to test for multicollinearity. The VIF values for all variables were less than 5, indicating the absence of multicollinearity in this regression model.

Abbreviations: aOR, adjusted odds ratio; CI, confidence interval; OAA/S scale, observer assessment of alertness/sedation scale; OR, odds ratio; TIVA, total intravenous anesthesia; WFNS, world federation of neurosurgery societies scale.

We then compared three derived variables (the AUC, the duration, and the TWA) of each prespecified level of MAP (Table 5). The differences in AUC and duration of 65, 70, and 75 mmHg between patients with and without newly developed cerebral infarction reached statistical significance in the univariate analysis, whereas the differences were not significant any more after adjustment. On the other hand, all three derived variables of 40%, 30%, and 20% decrease from baseline value were not only significantly associated with newly developed cerebral infarction in the univariate analysis (Table 5), but the AUC‐MAP and the TWA‐MAP of 30% decrease (both *p* = 0.04), and the AUC‐MAP (*p* = 0.02) and the TWA‐MAP of 20% decrease (*p* = 0.04) were also significantly associated with the newly developed cerebral infarction. A subgroup analysis demonstrated a statistically significant interaction between ASA physical status classification of ≥ III and TWA‐MAP decrease in 20% with postoperative newly developed cerebral infarction (*p* for interaction = 0.02), as shown in Figure S1.

**TABLE 5:** Associations between hypotension and newly developed cerebral infarction in univariate and multivariate analysis.

	Univariate analysis			Multivariate analysis		
	OR	95% CI	*p*	aOR	95% CI	*p*
AUC‐MAP (IQR), mmHg⋅min						
Below 65 mmHg	1.00	1.000–1.002	0.05	1.00	1.000–1.002	0.20
Below 70 mmHg	1.00	1.000–1.001	0.02	1.00	1.000–1.001	0.19
Below 75 mmHg	1.00	1.000–1.001	0.02	1.00	1.000–1.000	0.30
Duration (IQR), min						
Below 65 mmHg	1.01	1.002–1.011	0.01	1.01	1.000–1.009	0.06
Below 70 mmHg	1.00	1.000–1.006	0.04	1.00	0.998–1.004	0.45
Below 75 mmHg	1.00	1.000–1.004	0.05	1.00	0.998–1.003	0.71
Time weighted average MAP, mmHg/min						
Below 65 mmHg	1.16	0.935–1.443	0.18	1.19	0.945–1.474	0.14
Below 70 mmHg	1.10	0.984–1.217	0.10	1.09	0.979–1.215	0.12
Below 75 mmHg	1.19	0.953–1.474	0.13	1.04	0.979–1.111	0.19
AUC‐MAP (IQR), mmHg⋅min						
Below 60%	1.00	1.000–1.000	0.01	1.00	1.000–1.000	0.05
Below 70%	1.00	1.000–1.000	0.00	1.00	1.000–1.000	0.04
Below 80%	1.00	1.000–1.000	0.00	1.00	1.000–1.000	0.02
Duration (IQR), min						
Below 60%	1.01	1.002–1.009	0.00	1.00	1.000–1.008	0.05
Below 70%	1.00	1.003–1.005	0.00	1.00	1.000–1.004	0.05
Below 80%	1.00	1.002–1.005	0.00	1.00	1.000–1.003	0.06
Time weighted average MAP, mmHg/min						
Below 60%	1.02	1.004–1.040	0.02	1.02	1.000–1.037	0.05
Below 70%	1.02	1.005–1.029	0.01	1.01	1.001–1.025	0.04
Below 80%	1.02	1.006–1.029	0.00	1.01	1.001–1.024	0.04

*Note:* In the multivariate analysis, the hypotensive parameters are adjusted by modified Fisher scale Grade 3–4 and mean duration of anesthesia. The area under receiver operating characteristic curve for AUC‐MAP of 20%, 30% decrease models was both 0.57 (95% CI: 0.53–0.61), and Time weighted average MAP of 20%, 30% decrease models was 0.55 (95% CI: 0.51–0.59), 0.56 (95% CI: 0.52–0.60). For above four variables *p* < 0.05 in the Hosmer–Lemeshow test.

Abbreviations: aOR, adjusted odds ratio; AUC, area under curve; CI, confidence interval; IQR, interquartile range; MAP, mean arterial pressure; OR, odds ratio.

## 4 Discussion

In this retrospective cohort study, newly developed cerebral infarction was observed with a rate of 21.6% after aSAH surgery, closely associated with 20% or more decrease from baseline MAP and obviously associated with poor clinical outcomes.

A recent meta‐analysis including studies on CT and MRI examinations indicated a significant association between early brain infarction and increased poor outcome, with a risk ratio of 2.18 for mortality and 2.26 for poor outcome [20]. The incidence of newly infarction in the current study was 21.6%, higher than 14% reported in CT studies, and lower than 41% in MRI studies. Similar to our results, Kenji et al. reported the incidence of early cerebral infarction was 28% in SAH patients undergoing early embolization or clipping of ruptured aneurysms [7].

Cerebral infarction is the result of various pathways of neuronal damage and accounts for multiple initial brain injury. The brain after aSAH may experience global ischemia, blood–brain barrier breakdown, edema, microvascular dysfunction, and large vessel vasospasm, which lead to the development of cerebral infarction [20, 21]. Our study found that bleeding severity was independently associated with newly developed cerebral infarction, consistent with previous research [7, 8]. Besides, we found a significant association between anesthesia duration and postoperative newly developed cerebral infarction. In addition, intracranial hypertension, lobal impairment of cerebral perfusion, symptomatic, or evidence of vasospasm predict newly developed cerebral infarction [7, 8, 22]. Animal studies have shown that hemodynamic changes and severe intracranial hypertension are associated with a reduction in cerebral perfusion pressure during the early period [23]. However, our study did not retrospectively collect the other potential risk factors. Thus, maintaining hemodynamic stability and preventing cerebral ischemia are critical in perioperative management for high‐risk aSAH patients, especially to avoid hypotension‐induced cerebral ischemia [24].

CBF may maintain stable with MAP changing within the range between 70 and 150 mmHg for normal brain [25]. Therefore, the absolute threshold of hypotension is defined as MAP of 70 ± 5 mmHg in this study. For the relative thresholds, 20% decrease from baseline MAP is often maintained. A series of studies investigated the association between intraoperative blood pressure level and postoperative cerebral infarction with inconsistent results. A multicenter retrospective cohort study reported no association between 75 mmHg or relative 20% lower than baseline and major adverse cardiac and cerebrovascular events in the 30‐day postoperative period [26]. Our previous study evaluated the intraoperative hypotension in elderly patients who underwent resection of brain tumor and showed postoperative strokes were not associated hypotension, which was defined as MAP less than or equal to 70 mmHg [27]. However, a retrospective study found intraoperative hypotension (≥ 20% of baseline systolic blood pressure for ≥ 15 min) was an independent risk factor for the development of cerebral infarction in aSAH patients [28]. In the current study, the associations based on hypotension thresholds of 20% lower than baseline with postoperative ischemia was statistically significant. The function of automatic regulation in cerebral blood flow was impaired in aSAH animals and patients [29, 30], which suggested that intraoperative blood pressure management under general anesthesia was very important for patients. It seems evident that the mechanism of a postoperative newly developed cerebral infarction is multifactorial in nature. The primary cause is assumed to be cerebral thrombosis and insufficient cerebral perfusion [31]. Intraoperative hypotension may lead to insufficient cerebral perfusion which may increase the risk of newly developed cerebral infarction after operation.

The breakdown of blood–brain barrier, edema, and microvascular dysfunction after aSAH often leads to blood pressure increase to maintain cerebral perfusion pressure. From this point, the MAP level should be maintained based on the preoperative baseline value and should be applied the relative value. A subgroup analysis demonstrated the significantly statistical interaction between the TWA‐MAP decreases of 20% baseline and ASA physical status classification of ≥ III in the association with postoperative newly developed cerebral infarction, indicating that patients with poor physical status are particularly prone to postoperative newly developed cerebral infarction.

There are some limitations. Firstly, this was a retrospective cohort study without prospective collection of intraoperative blood pressure data, which could lead to measurement bias. However, the pressure data extracted cannot be altered and was the original continuous data. Secondly, we did not preestimate sample size, and the relatively limited sample size may contribute to the negative association between the thresholds of blood pressure and newly developed cerebral infarction. Thirdly, patients who underwent either clipping or coiling were included in the analysis. However, there were significant differences in two treatments procedures in terms of duration and intraoperative blood pressure. Finally, this was a single‐center retrospective cohort study and might not be generalizable or establish causality.

## 5 Conclusion

In this retrospective cohort study, the newly developed cerebral infarction was observed with a rate of 21.6% following aSAH after treatment, and independently associated with the level of MAP decreased 20% and more from baseline. Further multicenter randomized trial is needed to investigate whether the prevention from intraoperative hypotension reduces the risk of postoperative newly developed cerebral infarction after aSAH, especially for patients with poor physical status.

## Disclosure

The authors have nothing to report.

## Ethics Statement

This study was approved by the Medical Ethics review board of Beijing Tiantan Hospital (No. KY 2020‐11‐26).

## Consent

The Ethics Committee approved this study and waived the requirement for informed consent.

## Conflicts of Interest

The authors declare no conflicts of interest.

## Permission to Reproduce Material From Other Sources

This study did not reproduce other materials.

## Supporting information


Figure S1.

## Data Availability

Data are available upon reasonable request.
