# Supplementary figures and images for: Intraoperative Hypotension and Postoperative Newly Developed Cerebral Infarction in Patients With Aneurysmal Subarachnoid Hemorrhage: A Retrospective Cohort Study
Source: CNS Neurosci Ther. 2024 Dec 9;30(12):e70156. doi: 10.1111/cns.70156 (PMC11626475; doi:10.1111/cns.70156)

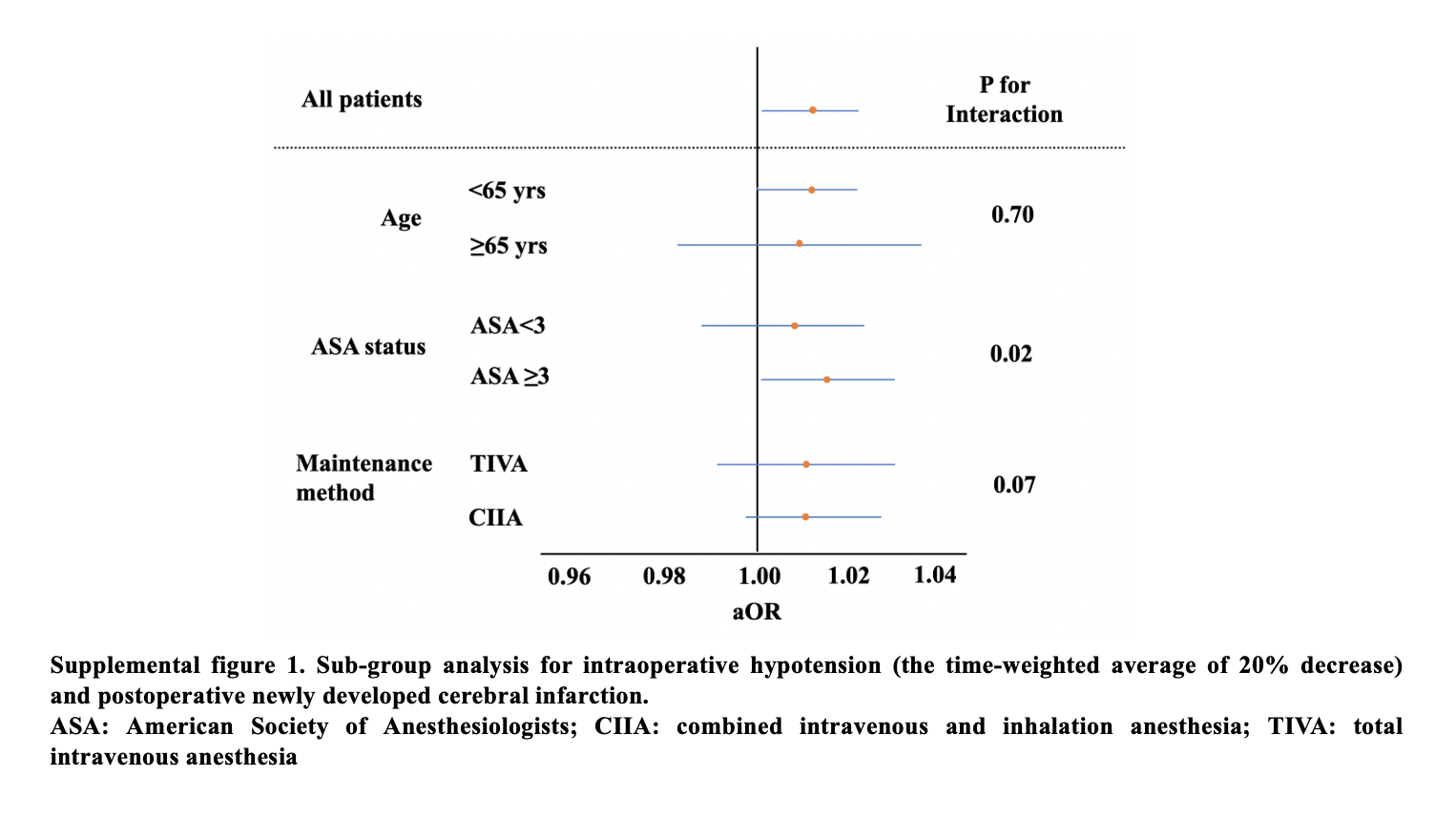

Supplement: Supplementary file 1 — Figure S1. [file CNS-30-e70156-s001.tiff]
